# Investigation of adaptive muscle synergy modulated motor responses to grasping perturbations

**DOI:** 10.1038/s41598-024-68386-8

**Published:** 2024-08-09

**Authors:** Eike Jakubowitz, Leonard Schmidt, Alina Obermeier, Svenja Spindeldreier, Henning Windhagen, Christof Hurschler

**Affiliations:** 1 Laboratory for Biomechanics and Biomaterials, Department of Orthopaedic Surgery, Hannover Medical School, Anna-Von-Borries-Strasse 1-7, 30625 Hannover, Germany; 2 Institute of Mechatronic Systems, Leibniz University Hannover, An Der Universität 1, 30823 Garbsen, Germany; 3 Department of Orthopaedic Surgery, Hannover Medical School, Anna-Von-Borries-Strasse 1-7, 30625 Hannover, Germany

**Keywords:** Muscle synergies, Object lifting, Motor control strategies, Grasping performance, External perturbation, Myoelectric arm prostheses, Motor neuron, Neuromuscular junction, Biomedical engineering

## Abstract

This study investigated how muscle synergies adapt in response to unexpected changes in object weight during lifting tasks. The aim was to discover which motor control strategies individuals use to maintain their grasping performance. Muscle synergies were extracted from the muscle activity of fifteen healthy participants who lifted objects of identical appearance but varying weights in a randomized order, which introduced artificial perturbations. Reaching and manipulation phases of object lifting were analyzed using constrained non-negative matrix factorization and k-means clustering. Participants exhibited a perturbation-independent and thus consistent recruitment of spatial synergy components, while significant adaptations in muscle synergy activation occurred in response to unexpected perturbations. Perturbations caused by unexpectedly heavy objects led to delayed and gradual increases in muscle synergy activation until the force required to lift the object was reached. In contrast, perturbations caused by lighter objects led to reductions in excess muscle synergy activation occurring later. Sensorimotor control maintains the modularity of muscle synergies. Even when external mechanical perturbations occur, the grasping performance is preserved, and control is adapted solely through muscle synergy activation. These results suggest that using pure spatial synergy components as control signals for myoelectric arm prostheses may prevent them from malfunctioning due to external perturbations.

## Introduction

Humans can dexterously grasp and manipulate very different types of objects. The complex movements (kinematics) required from the upper limb to achieve these are the outcome of the action of numerous muscles, and this involves at least 27 degrees of freedom (DOF; about 7 for the arm and 20 for the hand, including fingers). This large number of DOF allows multiple motion patterns to be derived for accomplishing the same single task and motion goal, which illustrates the considerable redundancy of the musculoskeletal system^1^. Nevertheless, adapting the motor behavior to objects of different weights, shapes, and surfaces, as well as to changing environments, occurs without having to limit the motor performance. Sensorimotor control functions significantly contribute to these adaptation processes. Both predictable (feedforward) and unexpected (feedback) sensory information are used to generate and shape motor performance^2,3^. Based on previous experiences (sensorimotor memory) and the expected physical properties of the object, motor control is predicted (feedforward) so that a time-dependent action is planned and executed, including the sensory consequence of the intended motion. This predictive motor control is complemented by a correction procedure based on the feedback, which allows the course of action to be monitored and successive motor instructions to be rapidly adjusted if required^4^.

However, greater motor correction needs may arise when misleading sensory cues cause fundamentally flawed predictions with incorrect action planning. This is the case, for example, when there is no logical relationship between the size and weight of an object. In serial lifting tests with interspersion of unexpected weight changes, the motor corrections could even be physically detected^5^. For example, if a heavier weight is followed by a lighter one, a comparatively higher grasping force, which is actually not required, was observed. Using functional MRI (fMRI) data, further studies have focused on investigating the origin of these motor changes in the brain^6,7^.

In addition to these findings, there is also the theory of muscle synergies for the brain^8^. It is based on the idea that the central nervous system (CNS) uses an alphabet of building blocks to generate the entire panorama of upper limb kinematics. These building blocks represent time-independent weights (motor modules) that are thought to be stored in the CNS. To generate specific muscle patterns for a grasping task these building blocks are first recruited as motor modules and then composed by activation patterns as time-dependent commands (motor primitives)^9,10^. It is further hypothesized that this will allow the large number of upper limb DOF to be managed in a reduced vector space, potentially leading to a physiological simplification of the motor control problem, which thus avoids the redundancy problem^11,12^. Using time-invariant spatial models^13–15^, it has so far been established that fixed muscle weights combined with their variable activation coefficients can be represented primarily based on non-negative matrix factorizations (NNMF)^16^, e.g. derived from electromyographic (EMG) measurements.

Notwithstanding ongoing controversies about possible applications – for example, in motion production, exoprosthetic control or motion classification – the analysis of muscle synergies provides a compact description of muscle patterns and has thus established itself as a useful approach for evaluating motor control strategies^17,18^. However, muscle synergies also particularly enabled investigations of motor control affected by perturbations in the lower extremities. For example, fewer leg muscle synergies are recruited during reactive stepping following a perturbation than during an unperturbed stepping^19^. It has also been shown that neuromotor control, including both spatial and temporal muscular synergies, is modulated during events such as landing on unstable versus stable surfaces after jumping^20^. In contrast, the composition of muscle synergies does not appear to change in a muscular synergy model during adaptation to a perturbation in repetitive motions, such as locomotion, but is probably modulated exclusively by their activation patterns^21^.

As extracted muscle synergies are increasingly explored for the control of myoelectric prostheses to enable individual, intuitive, and dexterous finger movements of artificial hands^22,23^, the question arises how perturbations might affect these systems. It is important to note that the time-varying command primitives are not used in such control systems, but only the local time-invariant weights^22^. Additionally, subject-invariant weights of muscle synergies are only partially task-specific, as muscle interactions can be task-independent, redundant, or complementary^24^. If this complex level of motor modules is involved in perturbation-induced modulation, it could have a significant impact on the performance of such control technologies.

However, for the upper limb, it is still unknown how perturbations due to flawed predictions of—for example—the object’s weight are compensated motorically and how the synergy model can modulate such a perturbation. Based on this model, four discerning modulation prospects can be astutely derived, thus concurrently serving as hypotheses: The modulation of the synergy model occurs via (1) a recruitment of less or additional motor modules, (2) an altered muscle weighting within the recruited motor modules, (3) an altered activation pattern for the recruited synergies, and (4) a combination of both altered muscle weighting and altered activation patterns.

Therefore, based on the muscle synergy model, the aim of this experimental study was to investigate how perturbations in the motor control system are compensated.

## Materials and methods

All procedures described in this study were approved by the Ethics Committee of the Hannover Medical School (no. 3364) and conducted in accordance with the Declaration of Helsinki. Fifteen healthy subjects S1-S15 (mean age = 44.5 ± 15.3 years; f:m = 6:9; all right-handed) were recruited. Inclusion criterion was an age range of 18 to 75 years. Exclusion criteria were upper limb mobility impairments, neurological abnormalities, and cognitive deficits. All subjects provided written informed consent.

### General procedure

**Figure 1:**
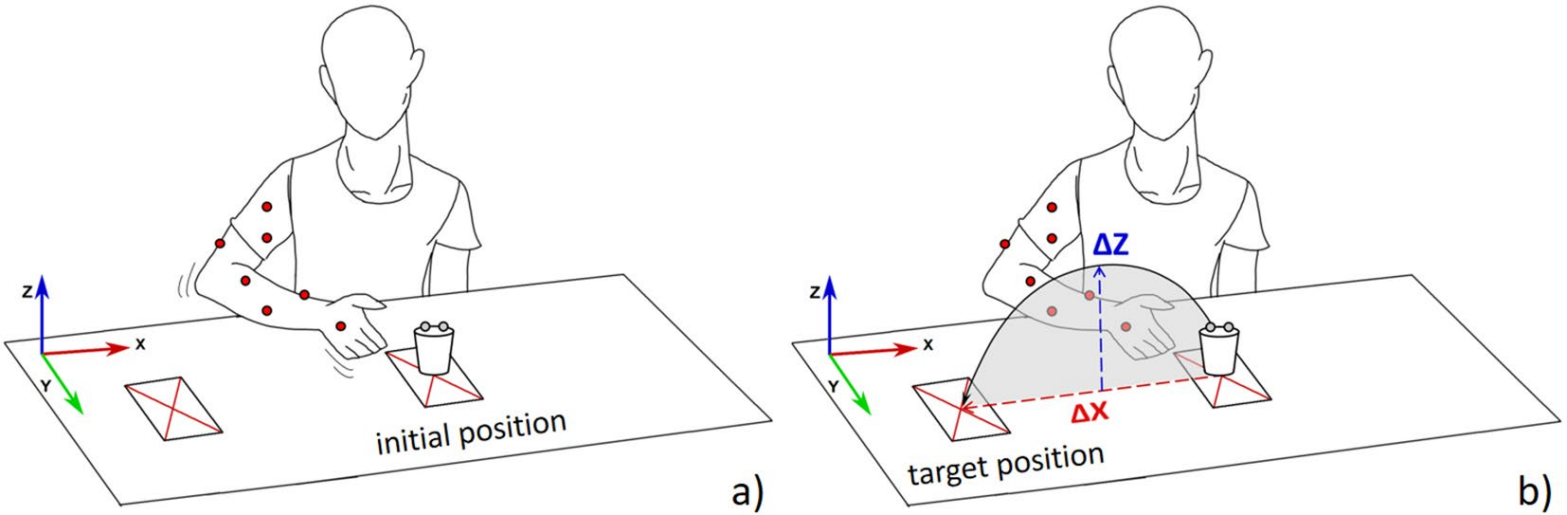
The participants were tasked with grasping the cup from the initial position and relocating it to the target position. Subsequently, the captured data sets were segmented to isolate the two distinct phases: reaching (**a**) and manipulation (**b**).

During experiments, subjects sat at a desktop with the marked initial position of the right hand and the initial and target positions of the object to be moved on the surface (Fig. 1). To ensure comparable kinematics, the desk was height-adjusted for each subject such that the right upper arm posture was approximately in 45° anteversion at the end of the reaching phase. Subjects were asked to perform the following grasping task repeatedly and fluently as soon as they received the auditory stimulus (“go”): to grasp a rigid plastic drinking cup positioned centrally on the desk, starting from the initial position of the hand, and place it 25 cm to the right onto the target position.

The task was performed at four weight increments obtained using cups filled with different amounts of sand (cup A = 50 g, B = 250 g, C = 450 g, and D = 650 g), similar to previous experimental studies^6,7,25^ to ensure sufficient perturbation intensity was generated during the experiments. The cups were presented to the subjects with n = 32 repetitions, so that neither the appearance (uniform and sealed cups) nor the noise generated during the placement on the desk (noise dampening pad) allowed for prior assessment of the weight of the cups by the subjects.

In the first phase of the experiment (random trial), the cups A, B, C, and D were randomly presented to the subjects with four repetitions each (n = 16). The second phase involved a block trial in which the cups were presented in four randomized blocks of the same weight, each with four repetitions (n = 16). All randomizations were generated anew for each subject using the random number array code in MATLAB (R2012a, MathWorks Inc., Natick, MA, USA). Subjects were not presented with any cues or knowledge about the type of experiment, weight, or increments. They were simply asked to move the cup.

### Data collection

The cups were fitted with two retroreflective marker balls (12mm) to track cup trajectories using a twelve camera motion capture system (Type MX, Vicon Motion System Ltd., Oxford, UK) and the corresponding Nexus software (Version 1.8.5) at a sampling rate of 200Hz. Videos were taken at the same sampling rate in the sagittal and frontal planes using digital video cameras (Pilot pi640gc, Basler, Ahrensburg, Germany) and synchronized with the MX system. A wireless surface EMG (sEMG) system (Trigno™ EMG, Delsys Inc., Natick, USA) was used to capture sEMG potentials sampled at 2 kHz for the following twelve upper and forearm muscles: deltoideus pars clavicularis (DC), biceps brachii (BB), triceps brachii (TB), flexor digitorum superficialis (FDS), extensor digitorum (ED), brachioradialis (BR), flexor carpi ulnaris (FCU), extensor carpi ulnaris (ECU), pronator teres (PT), flexor carpi radialis (FCR), abductor pollicis brevis (APB), and abductor digiti minimi (ADM). Electrodes were placed on the skin according to the Surface EMG for Non-Invasive Assessment of Muscles (SENIAM) guidelines^26^. Cross-talk artefacts from closely located adjacent muscle bellies were reduced by using reduced-size sensors (Trigno™ Mini) for the PT, FCU, APB, and ADM muscles. Prior to electrode placement, the skin was shaved and cleaned with ethanol to decrease electrical skin resistance. For later signal normalization all subjects performed two maximum voluntary contraction (MVC) tests, as previously described by Kendall et al.^27^, for each of the muscles investigated. In these tests, an investigator (A.O.) manually applied force resistance to achieve isometry.

### Data processing

Custom MATLAB (R2021a) offline code was employed to process and analyze all data collected. The mean trajectory of both markers on the cup served to determine the resultant 3D cup trajectory. Raw sEMG signals were initially hardware band-pass filtered (20–450Hz cutoff) to include just the frequency spectrum of desired muscle activities^19,28^. Signal processing was performed according to recent recommendations for muscle-synergy analysis^28^. Furthermore, in order to reduce adverse effects of skin motion artefacts, sEMG signals were digitally high-pass filtered (50 Hz cutoff, 6th order Butterworth filter, zero-phase shift)^14,15,28^. Subsequently, full-wave rectification linear envelope detection was performed using a low-pass filter (6 Hz cutoff, 6th order Finite Impulse Response (FIR), 100 ms window). The maximum value from both MVC tests of each muscle served for envelope normalizations^14^. Using defined object-related release and contact events^5^, both the reaching phase (from hand lifts off the desktop surface until digits contact the object) and the manipulation phase (from digits contact the object until the object fully contacts the desktop surface again) were segmented visually using both videos and temporally normalized to 100% duration^30^ by linear length normalization (LLN)^31^.

### Analysis of groups

Three distinct lifting situations were derived from the performed grasping task: (1) the object possesses the same weight as the preceding one (block trials); (2) the object is heavier than expected; (3) the object is lighter than expected. Accordingly, these lifting situations were defined as (1) no perturbation (np); (2) perturbation by a heavier object (ph); and (3) perturbation by a lighter object (pl). The initial lifting of each block (first occurrence of a specific cup weight) was excluded from the analysis to ensure no unintended perturbation within the np group during a block switch.

### Synergy extraction and analysis

The non-negative matrix factorization (NNMF) technique was employed^32,33^ to address the synergy model (Eq. 1)^29^. Utilizing the multiplicative update approach^16^, synergy components *w*_*i*_ and *h*_*i*_ were extracted through the reduction of a cost function (Frobenius norm) spanning 1000 iterations. This extraction process was reiterated 50 times to avoid local minima, retaining the reconstruction result with the highest approximation quality to the original data^14,15^. For the minimal number of muscle synergies *N* to be extracted, the coefficient of determination regarding the total explained variance of muscle activity must account for R^2^ ≥ 85%^33^. In addition, no further synergies were included in the model when the extraction yielded an increase of ΔR^2^ < 5% in the total explained variation^14^. Muscle weights within the spatial components *w*_*i*_ were normalized to a unit length to ensure the comparability of all synergy vectors between zero and one for the synergy analysis described below^15^. The temporal components *h*_*i*_ were scaled accordingly.

1 $$sEMG(t) = \sum\nolimits_{i = 1}^{N} {w_{i} h_{i} \left( t \right) + e}$$

A synergy weight of > 0.4 was considered significant^19^. Initially, group-specific (np, ph, pl) cluster centroids of subject-specific motor primitives were computed using k-means clustering with cosine similarity as the distance metric^14,15^. Each clustering process was repeated 100 times with 1000 iterations to mitigate the impact of local minima^15^. If a synergy cluster contains synergies from at least one-third of subjects, its centroids were defined as subject-invariant^15^. The resultant sets of group-specific (ph, pl) synergy centroids served as basic data for a further, albeit constrained, synergy extraction. During this matrix factorization, either the spatial synergy component *w*_*i*_ or the temporal synergy component *h*_*i*_ was fixed by being provided to the algorithms from their respective components gained from the np group (block trials), and therefore kept constant^21^. Consequently, the unfixed component was re-extracted as a new variable from the sEMG data, and R^2^ was recalculated to detect potential alterations in the temporal or spatial domains. A modulation by the unfixed but modified synergy component would thus be recognizable by a consistent reconstruction quality. Therefore, the similarity measures dot-product *DOT* for the synergy vectors (*w*_*i*_) and the Pearson’s correlation coefficient *ρ* for its activation component (*h*_*i*_) were calculated between extracted and re-extracted components to detect the degree of a possible synergy modification.

### Statistics

A Wilcoxon signed-rank test was used to compare the analysis groups np, ph, and pl for isolated time and peak data of cup trajectories. A Wilcoxon rank-sum test was performed for all other parameters like synergy similarity values and coefficients of determination. A *p*-value < 0.05 was considered significant, and Cohen’s d was calculated to define effect sizes.

## Results

### Object motion

**Figure 2:**
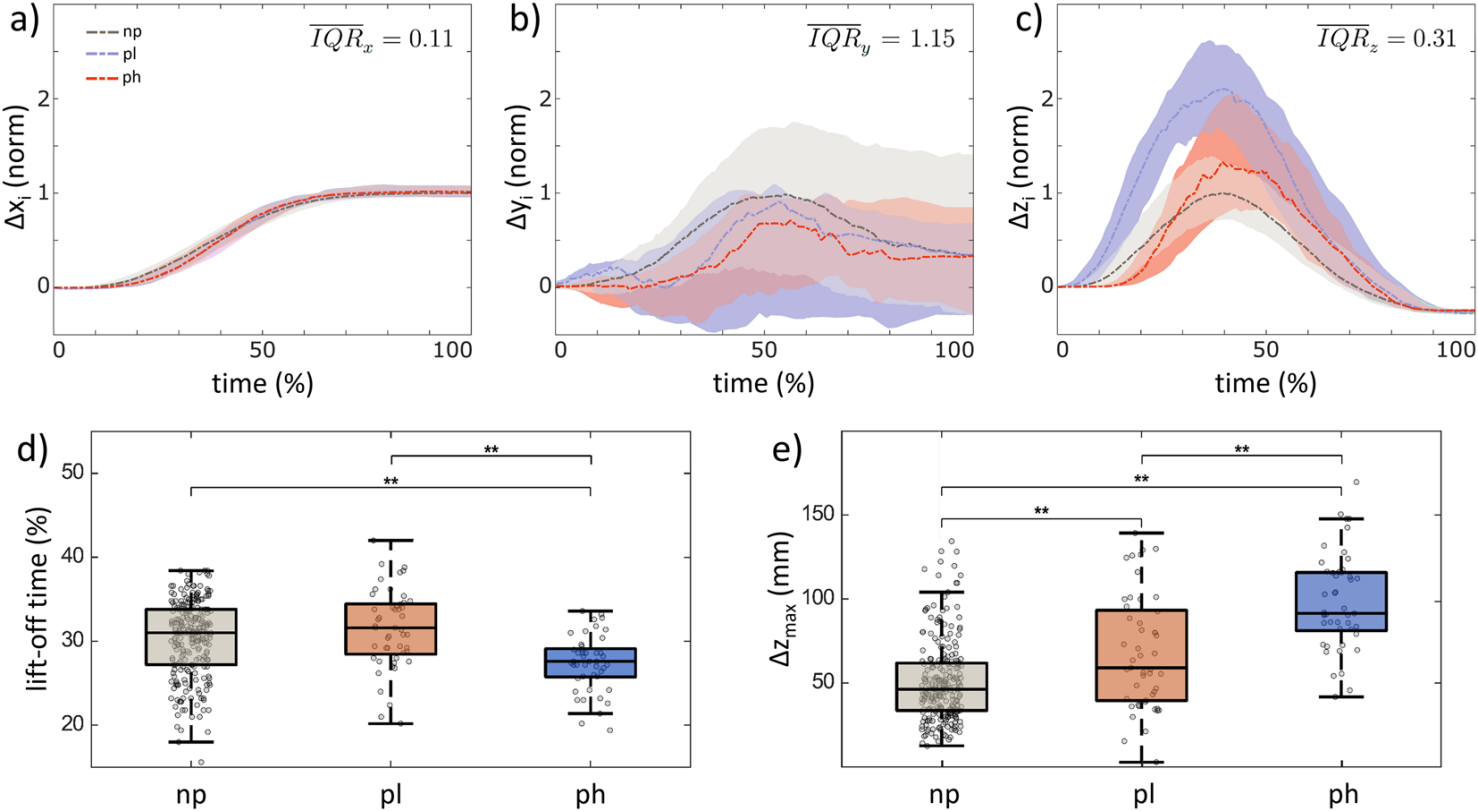
Medians and corresponding interquartile ranges *IQR* of the 3D object trajectories normalized to the unperturbed lifting situation (**a**–**c**); and box plots of the normalized lift-off time of the object (**d**) and the maximum absolute lifting height Δz_max_ (**e**). Lifting situations are shown as no perturbation (np) in black, the perturbation by a heavier object (ph) in red, and the perturbation by a lighter object (pl) in blue.

The object’s lateral motion from its starting to target position (x-trajectory, Fig. 2a) was consistent across all investigated groups (np = no perturbation, pl = perturbation by a lighter object, ph = perturbation by a heavier object), with minimal within-group variation. Conversely, the forward and backward motion (y-trajectory, Fig. 2b) exhibited significant within-group variation, but no differences were observed between the groups. With a moderate within-group variability, differences in group trajectories are clearly visible in the vertical object motion (z trajectory, Fig. 2c), as confirmed by both the temporal (Fig. 2d) and the geometric comparison (Fig. 2e). For example, the object leaves the desk surface significantly earlier in the pl group than in the np group. In contrast, a heavier object causes a significantly later lift-off from the desk surface compared to a lighter object. Nevertheless, the object is lifted significantly higher in the ph group than in the np group. When perturbed with a lighter object (pl), it is lifted more than twice as high as without perturbation (*p* < 0.01).

### Extracted synergies

With constant centroid numbers across all experimental groups, *K* = 5 clusters were found during the reaching and *K* = 7 clusters during the manipulation phase. Of these, *K* = 2 subject-invariant synergies were identified for the reaching phase, and *K* = 3 for the manipulation phase. During reaching, the APB muscle showed a significant involvement in the subject-invariant *w*_*1*_-synergy, whereas the DC muscle mainly contributed to the subject-invariant *w*_*2*_-synergy. The wrist muscles (FCU, ECU, FCR) and the finger extensor muscles (ED) contributed to a lesser extent to both synergies. The activation *h*_*2*_ was stronger in the early reaching phase, whereas *h*_*1*_ started at about 30% of the phase, reached its maximum at the end of the motion and then went into saturation. All similarity values *DOT* and *ρ* between the unperturbed and the perturbed groups were close to one, indicating high correlations.

**Figure 3:**
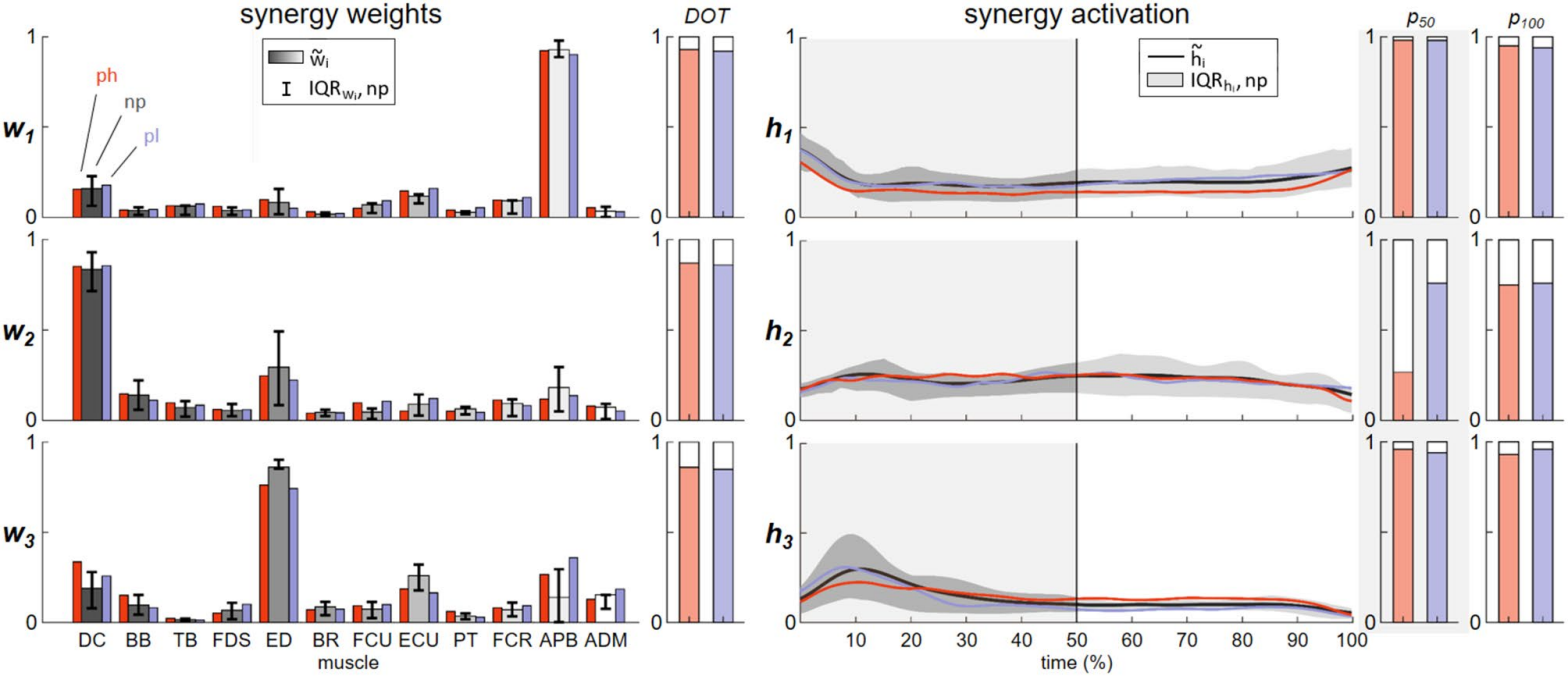
Subject-invariant median synergies with interquartile ranges (q_25_, q_75_) and ph and pl synergy centroids (bar graphs, left) and their median activations with interquartile ranges of the np activation (line graphs, right) during the manipulation phase for the analysis groups np (grey), ph (red), and pl (blue). The spatial (DOT) and temporal (p) similarity values for ph (red) and pl (blue) are shown next to each graph (p50 = p for the first 50% of the phase; and p100 = p for the entire phase).

During the manipulation phase (Fig. 3), the subject-invariant *w*_*1*_ and *w*_*2*_ synergy centers of the reaching phase remained active (significant contributions of the APB and DC muscles), with *w*_*2*_ also having a high weight of the ED muscle. The activation *h*_*1*_ decreased significantly after the first 10% of the phase and then remained constant. The finger and hand extensor muscles ED and ECU mainly formed the identified subject-invariant *w*_*3*_ synergy. Its activation *h*_*3*_ initially increased strongly, reached its maximum at 10% and then decreased again more slowly. The spatial components showed comparable muscle weights in all groups, although *w*_*3*_ had a higher weight on the APB muscle and a lower weight on the ED muscle in both perturbed groups. The temporal similarity values for *h*_*1*_ and *h*_*3*_ were comparable during the first half and the whole phase duration (*p*_*50*_ and *p*_*100*_ always close to one), whereas the activation of *h*_*2*_, especially in the first half of the manipulation phase, changed significantly in the ph group (*ρ*_*50*_ = 0.272) compared to moderate changes in the pl group (*ρ*_*50*_ = 0.762).

### Analysis of perturbations

**Figure 4:**
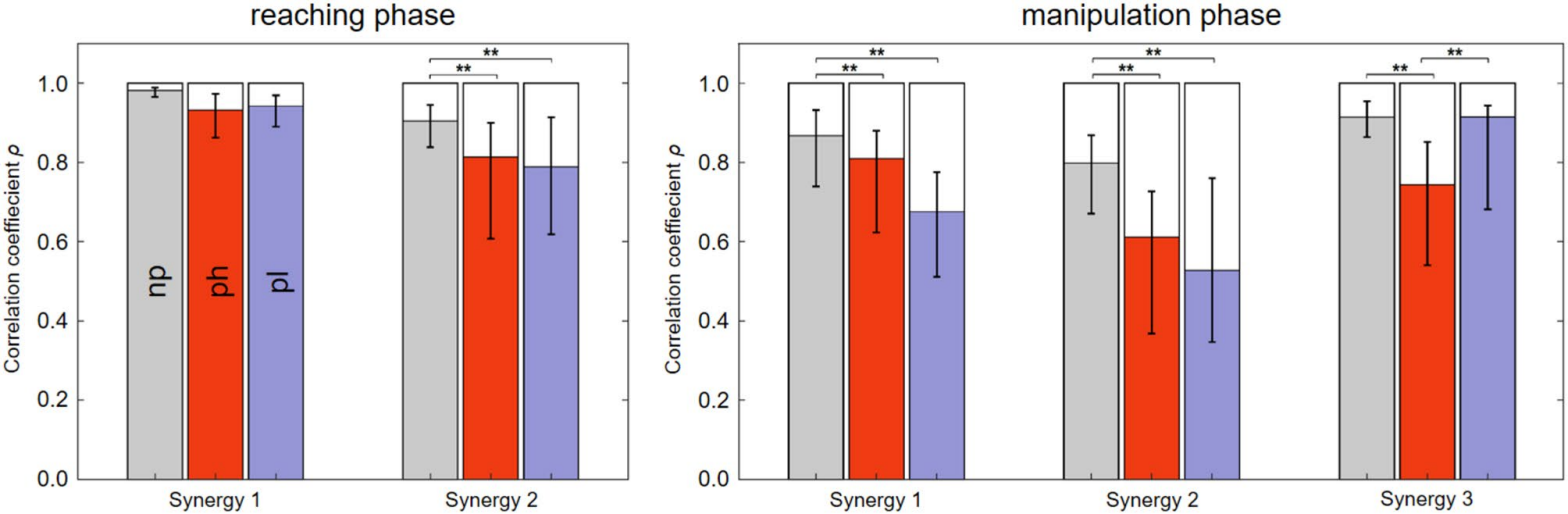
Cross-subject median and quantile of the temporal similarity value (*ρ*) for the groups np (grey), ph (red), and pl (blue) from the comparison between the synergy activations from the spatially constrained NNMF and the synergy extraction from the reference group np for the reaching phase (left) and the manipulation phase (right). Significance level: *p < 0.05; **p < 0.01.

The reconstruction of subject-invariant synergies using the spatially constrained NNMF for both the reaching and the manipulation phase does not lead to significant changes of R^2^ in the ph group (*p* = 0.06) and pl group (*p* = 0.32). Temporal synergy components after the spatial fixation show significant different synergy activations derived from both perturbed groups ph and pl compared to the reference synergy activations derived from the np group in all subjects (Fig. 4). Here, the activation profiles during reaching are less affected than those during manipulation. Within synergy 1 during reaching, the activations remained unchanged with similarity values of *ρ* ≤ 0.93 ≤ 0.98. However, a significant change in these similarity values can be observed for synergy 2 and both perturbation conditions (*p* < 0.01). During the manipulation, these similarity values are already significantly reduced in synergies 1 and 2 for both perturbation conditions (*p* < 0.01), whereas the changes in the pl group are always greater except for synergy 3. In this case, only the ph group differs significantly from the np group (*p* < 0.01), whereas the pl group does not differ. In contrast, the reconstruction using the temporally constrained NNMF resulted in a significant reduction of R_2_ in both groups with regard to the manipulation phase (*p* < 0.01 for ph; and *p* = 0.04 for pl).

## Discussion

### Summary of the main findings

The results show that additional motor modules do not appear to be recruited due to perturbations, so the first hypothesis is refuted. Furthermore, the results do not suggest that spatial synergy components can compensate for perturbations, so the second and fourth hypotheses are also rejected. However, the third hypothesis, an altered activation pattern for the recruited synergies, is confirmed.

Subject-invariant muscle synergies in perturbed grasping tasks, where the expected and actual object weight do not match, maintain their spatial structure and number but are modulated by temporally altered activations. These changes depend on the type of perturbation, are triggered by sensorimotor events, and thus occur during the feedback interval after the contact with the object is attained. In other words, an unexpected light object weight leads to an early object lifting from its base, whereas an unexpected heavy weight delays the object lifting. These adaptations correspond to the context-dependent nature of preprogrammed everyday actions requiring object-related force changes. The observed changes in the synergy activation also reflect necessary changes in force: a perturbation due to an unexpectedly light object leads to a delayed force reduction, while a perturbation by an unexpectedly heavy object leads to a gradual force increase until the object lifts.

Lastly, the results suggest that external perturbations probably do not impair the control of myoelectric prostheses, as only the motor modules serve as control signals and not their time-dependent activation patterns. This indicates that control based on motor modules should also be relatively robust against external perturbations. That is an important finding, as if motor modules do not have this robustness, using them as a control signal would be unreliable or potentially even dangerous.

### Detailed consideration of the findings

Clustering of recruited synergies identified two subject-invariant synergies during the reaching and three during the manipulation phases. The APB muscle contributes predominantly to the *w*_*1*_ synergy in both phases, whereas the DC muscle contributes to the *w*_*2*_ synergy. During reaching, *w*_*1*_ appears responsible for pre-shaping the hand for grasping the object, as it reaches its maximum activation around 50% of the phase, which then persists beyond the end of this phase, only dropping sharply shortly after the beginning of the manipulation phase, at around 10% of duration. The DC muscle in *w*_*2*_ is presumably responsible for transferring the hand to the object (lifting the arm and moving forward). The activation profile rises immediately during reaching and reaches its peak at around 20% of the reaching phase. Subsequently, activation moderately decreases, as the hand transfer experiences relatively strong acceleration initially, which then slows down as the hand approaches the object, resulting in deceleration.

These two synergies, along with a third, are also active throughout the manipulation phase. In the early part of this phase, *h*_*1*_ activation decreases rapidly (Fig. 3), as it is no longer necessary for pre-shaping the hand, and the hand has already adapted to the object size. In contrast, the *w*_*2*_ synergy remains active throughout the manipulation phase and appears to control the object transfer from the initial to the target position. The additional third synergy involves activation of the wrist and finger extensors (ED and ECU) and relatively small contributions of flexors (FCU and FCR). It is recruited exclusively during manipulation, suggesting its significant involvement in generating force for object grasping during the manipulation phase.

In general, the number of synergies required to account for the same variability in sEMG data under perturbed experimental conditions does not differ compared to the condition without perturbation. This holds true regardless of whether the recruited synergies possess subject-specific or subject-invariant characteristics. Therefore, it is highly unlikely that one or more additional synergies need to be recruited to compensate for perturbances caused by unexpected weight changes, refuting the first hypothesis. Additional motor modules do not appear to be recruited due to perturbations.

To identify which of the synergy components—motor modules or primitives—is responsible for the modulation of the perturbation, either the temporal or the spatial component of the pl- and ph-perturbed groups was methodically replaced by that of the reference group without perturbation during the constrained synergy extractions. A significant decline in the reconstruction quality would indicate whether changes caused by the perturbation could not be modulated by the respective unfixed and re-extracted synergy component. Therefore, the temporal-constrained synergy extraction examined whether there were changes in the spatial synergy components during the reconstruction of synergies for the object manipulation, which would confirm the second hypothesis. However, our results indicate that changes in sEMG patterns caused by unexpected weight changes cannot be reproduced by simply modifying the spatial component’s synergy weights while maintaining the temporal component constant. This suggests that the spatial components remain unchanged in large parts when the temporal components are fixed. Therefore, the vector space remains the same and is unaffected by the perturbation.

Conversely, the spatially constrained synergy extraction attempted to determine whether motor corrective responses occur over time during the object manipulation, which would correspond to the third hypothesis. It was found that the captured muscle activity during unexpected changes of the object weights could clearly be reconstructed by such a change. Both analysis parts confirm that the spatial components of synergies recruited during the reference experiments without perturbations could be kept constant, whereas the corresponding temporal activation was adapted for the compensation (Fig. 4). This confirms the third hypothesis for both perturbed conditions pl and ph, as well as for both phases of the grasping motion investigated here (reaching and manipulation). This result is consistent with the assumption of Cheung et al.^15^, who found that the spatial synergy model is time-invariant but is scaled by time-varying coefficients. Upper limb muscle synergies therefore appear to achieve resistance to external mechanical perturbations by adjusting only the temporal components *h*_*i*_ and thus the motor primitives. Severini et al. could also validate this for the lower limbs^14^. Motor adaptations in response to robot-induced motion perturbations similarly occur through temporal synergy components, whereas the motor modules remain constant in structure and number during repetitive leg motions.

The extent of temporally altered activation was assessed in this study using temporal similarity values *ρ*. A decrease in *ρ* during the reaching phase would have indicated that motor adaptations due to perturbations were driven by feedforward mechanisms, as no information about the object weight was available then. However, this was not the case. Rather, this decrease occurred especially in the second synergy during the first half of the manipulation phase. As this synergy likely plays a key role in object transfer, the decrease in similarity suggests a change in motor control based on feedback mechanisms. At this time, direct sensorimotor information about the object weight was available. Interestingly, this decrease is greater in perturbations caused by an unexpectedly higher object weight compared to perturbations caused by a lighter object weight.

If the temporal interval of this “overridden” activation (approximately 20–40% of the manipulation phase) is compared with the z-component of the object trajectory, it is immediately noticeable that the object does not leave the desktop surface until about 20% of the interval (lift-off time is significantly delayed). This suggests that sensorimotor feedback only occurred after this time and was subsequently followed by increased activation. As the force to lift the object was initially insufficient, the anticipated (feedforward) lifting from the surface did not occur. The observed reaction consists of a relatively long, gradual increase in force until vertical lifting is achieved, as Jenmalm^7^ also observed in his study. While the activation of the second synergy in this interval also differs in perturbations caused by an unexpectedly lighter object weight, it can be seen from the object trajectories that the object not only leaves the desktop significantly earlier but is also raised significantly higher. It seems that the excessively high force levels lead to earlier lifting with overshoot, and this unexpected sensorimotor event, in turn, leads to a cessation of the now excessive force levels^7^. Assuming the same interval for the duration of sensorimotor feedback as for perturbations caused by an unexpectedly heavier object weight (20% of the manipulation phase), the object is already raised one and a half times higher than in the situation without perturbation at 40% of the manipulation phase, reaching its maximum height. And precisely here, between 20 and 40%, a slight decrease in the activation profile of this synergy can be observed, explaining the decrease in similarity measure.

Interestingly, we observed that due to unexpected weight changes, there was a tendency towards altered activation of the second synergy even during the reaching phase, regardless of whether the weight was heavier or lighter. This could be because subjects may have gradually been prepared for weight changes due to the constantly changing weights, which could have influenced the feedforward mechanism from the outset.

### Limitations of the study

The methodology presented here and the resulting findings are, of course, subject to some limitations. One is that only a specific group of n = 12 muscles was included, which means the extracted synergies are only valid for this set of muscles. A different or larger set could have resulted in another spatial synergy structure. Nevertheless, it was shown with the extracted synergies that the muscles involved always exhibited a sufficient activity level above the noise level. Therefore, the robustness of muscle synergies was maintained even under the two perturbed conditions^29^. Conversely, it can even be stated that the extracted synergies suggest a lower activation level of muscles not included in this study. Additionally, the second synergy presented here was also identified in other studies as significant in the context of grasping tasks^34^ and can therefore be defined as reproducible across laboratories. Despite following the approach of other studies for the gradation of object weights^6,7,25^, these weight intervals can be considered another limitation, as it was unclear whether they led to the desired perturbation. However, we consider the validity of the experimental groups (np, pl, and ph) to be established through significantly different lifting times and trajectories of the object, allowing us to speak of valid analysis groups. Besides these experimental limitations, there are additional limitations resulting from assumptions about the synergy model and extraction algorithm used, as these are based on a linear spatial synergy model. Therefore, all results apply to the assumptions associated with this model, such as time-invariant spatial vector and time-variable synergy activation. However, as this is the most commonly used synergy model and ample experimental and behavioral evidence exists for its use^17^, we consider this a minor drawback.

## Data Availability

The datasets generated and/or analyzed during the current study are available from the corresponding author on reasonable request.

## References

[1] Bernstein, N. *The Coordination and Regulation of Movements* (Pergamon, 1967).

[2] Gallivan, J. P., Chapman, C. S., Wolpert, D. M. & Flanagan, J. R. Decision-making in sensorimotor control. *Nat. Rev. Neurosci.* **19**, 519–534. 10.1038/s41583-018-0045-9 (2018). 30089888 10.1038/s41583-018-0045-9 PMC6107066

[3] Edwards, L. L., King, E. M., Buetefisch, C. M. & Borich, M. R. Putting the “sensory” into sensorimotor control: The role of sensorimotor integration in goal-directed hand movements after stroke. *Front. Integr. Neurosci.* **13**, 16. 10.3389/fnint.2019.000162019 (2019). 31191265 10.3389/fnint.2019.000162019 PMC6539545

[4] Schneider, T. & Hermsdörfer, J. Anticipation in object manipulation: Behavioral and neural correlates. *Adv. Exp. Med. Biol.* **957**, 173–194. 10.1007/978-3-319-47313-0_10 (2016). 28035566 10.1007/978-3-319-47313-0_10

[5] Johansson, R. S. & Flanagan, J. R. Coding and use of tactile signals from the fingertips in object manipulation tasks. *Nat. Rev. Neurosci.* **10**, 345–359. 10.1038/nrn2621 (2009). 19352402 10.1038/nrn2621

[6] Schmitz, C., Jenmalm, P., Ehrsson, H. H. & Forssberg, H. Brain activity during predictable and unpredictable weight changes when lifting objects. *J. Neurophysiol.* **93**, 1498–1509. 10.1152/jn.00230.2004 (2005). 15385599 10.1152/jn.00230.2004

[7] Jenmalm, P., Schmitz, C., Forssberg, H. & Ehrsson, H. H. Lighter or heavier than predicted: Neural correlates of corrective mechanisms during erroneously programmed lifts. *J. Neurosci.* **26**, 9015–9021. 10.1523/JNEUROSCI.5045-05.2006 (2006). 16943559 10.1523/JNEUROSCI.5045-05.2006 PMC6675347

[8] Leo, A. *et al.* A synergy-based hand control is encoded in human motor cortical areas. *Elife* **5**, e13420. 10.7554/eLife.13420 (2016). 26880543 10.7554/eLife.13420 PMC4786436

[9] Mussa-Ivaldi, F. A., Giszter, S. F. & Bizzi, E. Linear combinations of primitives in vertebrate motor control. *Proc. Natl. Acad. Sci. USA* **91**, 7534–7538 (1994). 8052615 10.1073/pnas.91.16.7534 PMC44436

[10] Bizzi, E. & Cheung, V. C. The neural origin of muscle synergies. *Front. Comput. Neurosci.* **7**, 51. 10.3389/fncom.2013.00051 (2013). 23641212 10.3389/fncom.2013.00051 PMC3638124

[11] Bizzi, E., Cheung, V. C., d’Avella, A., Saltiel, P. & Tresch, M. Combining modules for movement. *Brain. Res. Rev.* **57**, 125–133. 10.1016/j.brainresrev.2007.08.004 (2007). 18029291 10.1016/j.brainresrev.2007.08.004 PMC4295773

[12] Hagio, S. & Kouzaki, M. Modularity speeds up motor learning by overcoming mechanical bias in musculoskeletal geometry. *J. R. Soc. Interface* **15**, 20180249. 10.1098/rsif.2018.0249 (2018). 30305418 10.1098/rsif.2018.0249 PMC6228487

[13] Israely, S., Leisman, G., Machluf, C. C. & Carmeli, E. Muscle synergies control during hand-reaching tasks in multiple directions post-stroke. *Front. Comput. Neurosci.*10.3389/fncom.2018.00010 (2018). 29527159 10.3389/fncom.2018.00010 PMC5829096

[14] Scano, A. *et al.* A comprehensive spatial mapping of muscle synergies in highly variable upper-limb movements of healthy subjects. *Front. Physiol.* **10**, 1231. 10.3389/fphys.2019.01231 (2019). 31611812 10.3389/fphys.2019.01231 PMC6777095

[15] Cheung, V. C. K. *et al.* Plasticity of muscle synergies through fractionation and merging during development and training of human runners. *Nat. Commun.* **11**, 4356. 10.1038/s41467-020-18210-4 (2020). 32868777 10.1038/s41467-020-18210-4 PMC7459346

[16] Lee, D. D. & Seung, H. S. Learning the parts of objects by non-negative matrix factorization. *Nature* **401**, 788–791. 10.1038/44565 (1999). 10548103 10.1038/44565

[17] Cheung, V. C. K. & Seki, K. Approaches to revealing the neural basis of muscle synergies: A review and a critique. *J. Neurophysiol.* **125**, 1580–1597. 10.1152/jn.00625.2019 (2021). 33729869 10.1152/jn.00625.2019

[18] Berger, D. J., Masciullo, M., Molinari, M., Lacquaniti, F. & d’Avella, A. Does the cerebellum shape the spatiotemporal organization of muscle patterns? Insights from subjects with cerebellar ataxias. *J. Neurophysiol.* **123**, 1691–1710. 10.1152/jn.00657.2018 (2020). 32159425 10.1152/jn.00657.2018

[19] Wang, S., Varas-Diaz, G. & Bhatt, T. Muscle synergy differences between voluntary and reactive backward stepping. *Sci. Rep.* **11**, 15462. 10.1038/s41598-021-94699-z (2021). 34326376 10.1038/s41598-021-94699-z PMC8322057

[20] Munoz-Martel, V., Santuz, A., Bohm, S. & Arampatzis, A. Proactive modulation in the spatiotemporal structure of muscle synergies minimizes reactive responses in perturbed landings. *Front. Bioeng. Biotechnol.* **9**, 761766. 10.3389/fbioe.2021.761766 (2021). 34976964 10.3389/fbioe.2021.761766 PMC8716881

[21] Severini, G. *et al.* Robot-driven locomotor perturbations reveal synergy-mediated, context-dependent feedforward and feedback mechanisms of adaptation. *Sci. Rep.* **10**, 5104. 10.1038/s41598-020-61231-8 (2020). 32214125 10.1038/s41598-020-61231-8 PMC7096445

[22] Yeung, D. *et al.* Co-adaptive control of bionic limbs via unsupervised adaptation of muscle synergies. *IEEE Trans. Biomed. Eng.* **69**, 2581–2592. 10.1109/TBME.2022.3150665 (2022). 35157573 10.1109/TBME.2022.3150665

[23] Yağmur Günay, S., Quivira, F. & Erdoğmuş, D. Muscle synergy-based grasp classification for robotic hand prosthetics. *Int. Conf. Pervasive. Technol. Relat. Assist. Environ.* **2017**, 335–338. 10.1145/3056540.3076208 (2017). 31111121 10.1145/3056540.3076208 PMC6525615

[24] Ó’Reilly, D. & Delis, I. Dissecting muscle synergies in the task space. *eLife* **12**, 87651. 10.7554/eLife.87651.3 (2023). 10.7554/eLife.87651.3 PMC10942626 38407224

[25] Johansson, R. S. & Westling, G. Coordinated isometric muscle commands adequately and erroneously programmed for the weight during lifting task with precision grip. *Exp. Brain. Res.* **71**, 59–71. 10.1007/BF00247522 (1988). 3416958 10.1007/BF00247522

[26] Hermens, H. J. *et al.**European Recommendations for Surface Electromyography: Results of the Seniam Project (SENIAM)* (Roessingh Research and Development, Enschede, 1999).

[27] Kendall, F. P. *et al.**Muscles: Testing and Function with Posture and Pain* (Lippincott Williams & Wilkins, 2001).

[28] De Luca, C. J., Gilmore, L. D., Kuznetsov, M. & Roy, S. H. Filtering the surface EMG signal: Movement artifact and baseline noise contamination. *J. Biomech.* **43**, 1573–1579. 10.1016/j.jbiomech.2010.01.027 (2010). 20206934 10.1016/j.jbiomech.2010.01.027

[29] Turpin, N. A., Uriac, S. & Dalleau, G. How to improve the muscle synergy analysis methodology?. *Eur. J. Appl. Physiol.* **121**, 1009–1025. 10.1007/s00421-021-04604-9 (2021). 33496848 10.1007/s00421-021-04604-9

[30] Lencioni, T. *et al.* A randomized controlled trial on the effects induced by robot-assisted and usual-care rehabilitation on upper limb muscle synergies in post-stroke subjects. *Sci. Rep.* **11**, 5323. 10.1038/s41598-021-84536-8 (2021). 33674675 10.1038/s41598-021-84536-8 PMC7935882

[31] Helwig, N. E., Hong, S., Hsiao-Wecksler, E. T. & Polk, J. D. Methods to temporally align gait cycle data. *J. Biomech.* **44**, 561–566. 10.1016/j.jbiomech.2010.09.015 (2011). 20887992 10.1016/j.jbiomech.2010.09.015

[32] Cheung, V. C., d’Avella, A., Tresch, M. C. & Bizzi, E. Central and sensory contributions to the activation and organization of muscle synergies during natural motor behaviors. *J. Neurosci.* **25**, 6419–6434. 10.1523/JNEUROSCI.4904-04.2005 (2005). 16000633 10.1523/JNEUROSCI.4904-04.2005 PMC6725265

[33] Rabbi, M. F. *et al.* Non-negative matrix factorisation is the most appropriate method for extraction of muscle synergies in walking and running. *Sci. Rep.* **10**, 8266. 10.1038/s41598-020-65257-w (2020). 32427881 10.1038/s41598-020-65257-w PMC7237673

[34] Scano, A., Chiavenna, A., Malosio, M., Molinari Tosatti, L. & Molteni, F. Muscle synergies-based characterization and clustering of poststroke patients in reaching movements. *Front. Bioeng. Biotechnol.* **5**, 62. 10.3389/fbioe.2017.00062 (2017). 29082227 10.3389/fbioe.2017.00062 PMC5645509

